# Older Migrant Patients and Health Care Professionals’ Experiences With Digital Translation Tools in Care Interactions: A Qualitative Literature Review

**DOI:** 10.1177/10436596241297644

**Published:** 2024-11-13

**Authors:** Sirpa Rosendahl, Viveca Larsson

**Affiliations:** 1 University of Skövde, Sweden

**Keywords:** caring context, culture, digital translation, older migrants, qualitative

## Abstract

**Introduction:**

Finding solutions to communicate difficulties in care interactions between health care professionals and older migrant patients may be facilitated by the use of digital translation tools. The aim was to explore older migrant patients’ and health professionals’ experiences using digital translation tools in transcultural care.

**Methodology:**

A systematic qualitative literature review, based on nine quality assessed articles published 2009 to 2024 from five databases, and analyzed using thematic analysis.

**Results:**

Three themes and eight subthemes emerged: *advantages of using translation tools* describes the benefits experienced using such tools; *limitations and challenges* highlights the problems identified in their use; and *improvement suggestions for the functions of the translation apps* describes adjustments and developments of the translation tools.

**Discussion:**

Digital translation used in basic care, may enhance relationships and equity of care, but should not substitute human interpreters in complex care conversations. Translation tools need to be developed according to older users’ abilities.

## Background

In care interactions in a multicultural world, it is important for health professionals and patients to understand each other culturally and linguistically to be able to provide high-quality care. An important part of nursing care is communication, as it is essential for care to be perceived as equitable. However, communication is not always the case when it comes to older migrant patients, as those with low language proficiency compared to the native population are at risk of being in a vulnerable position. “Older migrant patients” in this study refers to those who were born outside the hosting country or country of residence, are 60+, and speak languages other than the official language of the host country.

Studies have shown that health professionals communicate less with patients with low language proficiency, and they often experience lower quality of care (Ahmed et al., 2017; Derose et al., 2007). The consequences of not being able to understand and communicate with each other linguistically can result in misunderstandings regarding medical assessment, treatment, and care, and even in care injuries (Chang et al., 2021; Pietilä Rosendahl et al., 2016). Moreover, it can create anxiety and uncertainty in migrant patients during contacts with health care services (Harrison et al., 2020). There is also a risk that recovery after an illness will be prolonged (Karliner et al., 2010). Health professionals often use either authorized interpreters (Butow et al., 2012), family members of the patient, or bilingual health professionals when communicating with migrant patients (Graaf et al., 2012). In situations where no human interpreters are available, health professionals try to communicate using simple words or nonverbal signs (i.e., gestures, facial expressions, or pictures; Graaf et al., 2012; Söderman & Pietilä Rosendahl, 2016). As contemporary society and health care contexts becomes digitalized, digital tools to support communication in the care interactions are needed.

## Digital Communication Tools

Today, there is a wide range of digital tools that have been developed to support and facilitate health care (Kelley et al., 2011; Turjamaa et al., 2019). There are also digital tools that translate languages between patients and health professionals which can be used when no human interpreters are available (Michalec et al., 2015; Panayiotou et al., 2019). Digital translation tool in this study refers to machine translation with software which translates a written or orally spoken text from one language into another language. They can be downloaded onto a mobile phone, tablet, or computer and can be used online; for example, through web browsers, and offline through applications (apps). Translation tools used in health care contexts can use words in different languages with a collection of phrases sorted into categories, and the user can choose the most relevant one for the situation (Freyne et al., 2015; Panayiotou et al., 2019). Some applications only have one-way communication, while others have features for structured questions and answers available to the user. The user can ask a question and provide an answer orally, and the application translates the sentence to another language, or the message can be expressed in written words (Panayiotou et al., 2019).

The usability of a digital translation tool is always important, and particularly when it comes to older users. Due to age-related changes, older individuals may have impaired vision or hearing or age-related changes in cognitive functions, such as decreased ability to focus, concentrate, or decreased abilities to solve complex problems. This means that older individuals may require more time to learn how to use technical devices (Johnson & Finn, 2017). Earlier research has shown that older individuals are not unwilling—nor are they incapable of learning—to use technical tools, but rather that the number of older individuals using such technology has increased (Kim et al., 2022; Offerman et al., 2024). Moreover, as many countries’ current aging populations are multicultural, it should not be taken for granted that all older migrant adults can read or write in the majority language of their country of residence.

## Health Literacy

Poor language skills and low print health literacy, as communication barriers, have been linked to health outcomes (Tsoh et al., 2016). Health literacy is related to autonomy, decision-making, and empowerment, which is also the case for digital communication (Nutbeam, 2019; 2000). An important aspect of health literacy is to be able to make informed choices and decisions, on medical issues, risk factors for health etc. (Sørensen et al., 2012; World Health Organization [WHO], 2013). For health professionals to make informed decisions on treatment and care, and for patients to make informed choices for their health and medical needs, it is essential to understand each other in care interactions. Health literacy can also be associated with the language, values, behaviors and attitudes of a culture (Leininger, 1990) and nurses need to be aware of that migrant patients receiving care are influenced by their cultural context (McFarland & Wehbe-Alamah, 2019).

Aging humans require human interaction, but when being dependent on others they are also vulnerable. When languages hinder good communication, the vulnerability of the older migrant patients, and the professionalism of the health professionals offering care may be affected. Digital tools can be used to overcome communication barriers when human interpreters are unavailable. However, both patients and health professionals need to be able to use these tools. Research on the perspectives of older migrant users of digital translation tools in care interactions with health professionals needs to be illuminated.

## Aim

To explore older migrant patients’ and health professionals’ experiences using digital translation tools in transcultural care.

## Method

This systematic literature review is based on qualitative, mixed-method, and case studies, to capture the experiences of the participants. Inclusion criteria were: older migrant patients approx. 60 years of age and above, who did not speak the language of the hosting country, and with ethnic origins in any country, health care professionals all ages and disciplines, digital translation tools used in a caring context, publication years 2007 to 2024. Exclusion criteria were: digital tools only consisting of pictures, quantitative studies and publication years before 2007, reviews and conference papers. Databases such as Cinahl, PubMed, Medline, Web-of-Science, and Scopus were used for data collection. Articles were analyzed using a thematic analysis inspired by Braun and Clarke (2006).

## Data Collection

The search terms used stem from the aim were: digital tools, language/translation, culture, older migrants, and caring context. The search terms were: (digital* OR digiti* OR comput* OR online* OR internet* OR smartphone* OR iphone* OR mobile* OR cellphone* OR “cell phone” OR “cell phones” OR “cellular phone” OR “cellular phones” OR tablet* OR ipad* OR software* OR app OR apps OR application OR applications OR eHealth OR “e-health” OR “e health” OR “electronic health”) **AND** (languag* OR lingu* OR speak* OR speech* OR translat* OR interpret* OR communicat* OR multiling* OR biling*) **AND** (transcultural* OR “trans-cultural” OR intercultural* OR “inter-cultural” OR crosscultural* OR “cross-cultural” OR “non-native” OR “non native” OR “foreign-born” OR “foreign born” OR immigra* OR migrant* OR migrat* OR refugee* OR “limited English proficiency” OR “limited English proficient” OR LEP OR “non English speaking” OR “non-English speaking” OR CALD OR “Culturally and linguistically diverse” OR NESB OR “non-English speaking background” OR “non English speaking background” OR “second language” OR “language barrier” OR “language barriers” OR “communication barrier” OR “communication barriers” OR multiling* OR biling*) **AND** (aged* OR elder* OR old* OR gero* OR geri*) **AND** (health care OR care OR caring OR nurse* OR nursing OR “group home” OR “group homes” OR “Assisted Living Facility” OR “Group Home” OR “Halfway House” OR “Assisted Living Facilities” OR “Group Homes” OR “Halfway Houses”). An initial search was conducted May 31, 2022 and an update was done June 13, 2024. The search of the articles 2024 yielded a total of 4,939 and 2434 duplicates were identified and removed. The remaining 2505 were screened for title and abstract and 27 were considered for inclusion.

**Figure 1.**
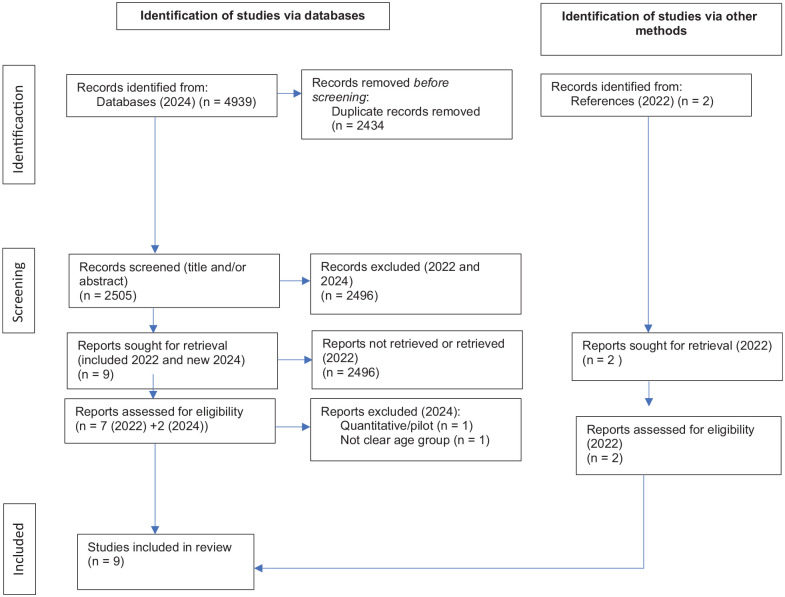
Selection of Articles, PRISMA Flowchart.

After the scientific quality assessment using JBI Critical Appraisal Checklist for Qualitative Research (Lockwood et al., 2015), nine articles were included in the study. The selection of articles is presented in a modified PRISMA flow chart (Page et al., 2021; see Figure 1).

Neither of the included articles met all JBI, criteria, but due to few qualitative studies, we decided to include all articles.

## Analysis

The articles were analyzed inspired by Braun and Clark’s (2006) analytical framework. First, all articles were read by the authors (SR/VL) to familiarize and get an overview with the article contents. Second, the text sections that addressed the purpose of this study in the articles’ results were identified. Third, the selected text sections were coded. Fourth, the codes were grouped together based on similarities and differences in the groups’ content. Fifth, preliminary themes were checked regarding the relationship between the themes and their respective subthemes. The sixth step included a description of the analysis, presented in the results section. Three articles were analyzed separately by both authors (SR/VL; step 2–4). All articles were analyzed by the first author (SR) and both authors discussed and compared the preliminary themes. To reach mutual consensus themes were reworked.

## Ethical Considerations

The included articles have been reviewed in light of relevant ethical guidelines. Seven of the included studies had received ethical permissions, and two of the studies elaborated upon ethical considerations and reflections.

## Results

The studies were conducted in Australia (5), United States (1), Northern Ireland (1), Japan (1), and Holland (1) during the years 2009 to 2022. The number of participants (both patients and health professionals) ranged from 1 (a case-study) to 134. The ages of the older migrant patients were approx. ≥60 years and they identified with 18 ethnic groups: Italian, Greek, Arabic, Vietnamese, Serbian, Cantonese, Macedonian, Spanish, Chinese, Bosnian, Croatian, Mandarin, Polish, Punjabi, Samoan, Bulgarian, Turkish–Dutch, and Moroccan-Dutch. Health professionals were from various disciplines, such as nurses, doctors, dieticians, and physiotherapists of all ages. The qualitative analysis methods employed included thematic content analysis, focus group methods, and inductive content analysis.

The translation tools in the chosen articles were CALD Assist, Talk-to-me, Google Translate (GT), and Health Communicator (Table 1). The concept “culturally and linguistically diverse” (CALD) is often used in Australia to describe those who were born overseas, speak languages other than the official national languages and/or have lower language levels in the national languages of their country of residence, and/or who have parents who were born overseas, and is meant to capture diversity in a broad sense (Australian Institute of Health and Welfare, 2018). CALD Assist and Talk-to-me are translation apps consisting of a phrase library with carefully selected health care-related phrases translated into a target language. Only simple health care–related phrases can be translated in the app, as this is expected to minimize the risk of misunderstandings. The phrases can be played audibly or seen visually through translated text or images (Panayiotou et al., 2020; Silvera-Tawil et al., 2018). GT was also used in the articles, as this translation tool already exists and is often used in health care conversations spontaneously. GT uses free text and can translate over 90 languages (Panayiotou et al., 2020). Health Communicator is a multilingual eHealth tool which provides structured questions for the migrant patient regarding his or her medical history. The tool is used as preparation for a medical consultation and patients can listen to or read the questions in this context (Yilmaz et al., 2020).

**Table 1.** Characteristics of Included Studies.

Author/year	Aim	Participants	Ethnic group	Location	Methods	Level of evidence
Freyne et al. (2018). Developing Digital Facilitation of Assessments in the Absence of an Interpreter: Participatory Design and Feasibility Evaluation with Allied Health Groups	To design and evaluate CALD Assist, a tablet to assist communication between patients and allied health clinicians in the absence of an interpreter	Health care staff (*n* = 19) Patients (*n* = 1)	The CALD Assist contained 10 languages	Australia	Mixed-method/The app ‘Cald Assist’ was used as a communication tool. Data consist of questionnaires/focus groups. Qualitative data consist of free-text responses of a quantitative questionnaire. The free-text responses were analyzed with NVivo computer program.	JBI: 4 yes, 2 no,4 unclear
Hwang et al. (2022). Testing the use of translation apps to overcome everyday health care communication in Australian aged-care hospital wards—An exploratory study.	**Aim:** To trial three mobile translation apps in the health care setting to address language barriers in everyday care between health care staff and older people with limited English proficiency (LEP).	Nursing and allied health staff (*n* = 24) and (*n* = 24) patients participated.	Italian Greek Arabic Vietnamese Serbian Cantonese Macedonian Spanish	Australia	A mixed-methods study. Three translation apps (CALD Assist, Talk to Me, Google translate) were used and the study was conducted across four aged-care hospital wards. Data were analyzed using descriptive statistics and thematic content analysis of open-ended responses in the surveys and observations.	JBI: 6 yes, 1 no, 3 unclear
Leyden & Melby (2021). Enteropathy-associated T-cell lymphoma manifesting as nonspecific abdominal pain: A case report highlighting the dangers of relying on Google Translate for clinical history taking	Explore how the communication was challenged between a doctor and a patient by a language barrier.	Patient, male, 60 years of age, Health professional (doctor) age unknown	Bulgarian	Northern Ireland	Case-study and Google translate was used as a communication tool in comparison to an authorized human translator.	JBI: 2yes,6 no,2 unapplicable
Panayiotou et al. (2020). The perceptions of translation apps for everyday health care in health care workers and older people: A multimethod study.	To understand the attitudes and perceptions of older people with limited English proficiency (LEP) and health care workers to using mobile translation technology for overcoming language barriers in the health care setting.	Older (*n* = 12) migrant adults, 65+ and health care staff (*n* = 17) age unknown	Greek Chinese	Australia	Multimethod design/focus groups with older migrant patients and focus groups with nurses and allied health professionals. Translation apps Cald Assist, Talk To Me and Google translate were used in the study. Qualitative data were analyzed using inductive content analysis.	JBI: 7yes,1no,2 unclear
Silvera-Tawil et al. (2018). CALD Assist—Nursing: Improving communication in the absence of interpreters	To develop a communication app to support nursing staff during the provision of standard care of patients from non-English-speaking backgrounds (NESBs), when an interpreter is not available.	Health staff (*n* = 15) in four focus groups and patients (*n* = 10) age 60+	Vietnamese, Greek, Italian Spanish.	Australia	A mixed-method study, based on focus groups, observations and interviews, with a quantitative component from observational data and staff surveys. A communication app; CALD Assist, which includes 95 commonly used phrases interpreted into 10 languages and grouped by discipline was used. Observations of interactions between health staff and patients.	JBI: 4 yes, 5 no,1 unclear.
Silvera-Tawil et al. (2021). Enabling Nurse-Patient Communication with a Mobile App: Controlled Pretest–Posttest Study with Nurses and Non-English-Speaking Patients	To evaluate the efficacy of the communication app to support nursing staff during the provision of standard care to patients from non-English-speaking backgrounds when an interpreter is not available	Patients (*n* = 7) age 48–90 years and nursing staff (*n* = 9) age unknown health staff (*n* = 92) for the surveys	Bosnian Cantonese Croatian English Greek Italian Spanish Macedonian Mandarin Polish Punjabi Samoan Serbian Vietnamese	Australia	A mixed-methods study. Data consists of 134 observations of nurse-patient interactions and 396 documented sessions of using the Cald Assist app and focus group interviews. The qualitative data was analyzed using a thematic analysis The quantitative material consists of surveys with nursing staff, which was analyzed with statistics.	JBI: 8 yes, 1 no, 1 unclear
Tatemoto et al. (2021). Overcoming language barriers to provide telerehabilitation for COVID-19 patients: A two-case report	Presents two cases of successful telerehabilitation delivery for patients quarantined due to COVID-19. One of the patients did not speak the therapist’s language.	2 patients 72 years old respective 49 years old Health professional, physiotherapeutic (*n* = 1) age unknown	One Cantonese speaking patient The other a deaf person	Japan	Case-study where Google Translate was used as a communication tool	JBI. 5 yes, 4 no, 1 unclear
Yilmaz et al.(2020) Enhancing patient participation of older migrant cancer patients: needs, barriers, and eHealth.	1. To gain insight into the unfulfilled instrumental and affective needs of Turkish-Dutch patients/survivors.2 the barriers perceived by health care professionals in fulfilling these needs and 3. How the Health Communicator, a multilingual eHealth tool, can support the fulfillment of patients’/ survivors’ needs, and decrease professionals’ barriers	Patients: *n* = 10, age approx. 70 year, *n* = 9, approx. 70 years Professionals: GP:s *n* = 7, approx. 45 years Oncological nurses, *n* = 5 Approx. 50 years	Turkish-Dutch Morrocan-Dutch Dutch non-Dutch	Holland	Qualitative/Focus groups and individual interviews when using the communication tool ‘Health communicator’.	JBI: 8 yes, 1 no, 1 unclear
Yost et al. (2009). Bilingual health literacy assessment using the Talking Touchscreeen/la Pantalla Parlanchina: Development and pilot testing	To describe the development and pilot testing of a new health literacy assessment tool	English-speaking (*n* = 97) and Spanish-speaking (*n* = 134) patients	Spanish English	USA	Mixed-method study ‘Talking Touch Screen’ was used as a communication tool	JBI: 8 yes, 1 no, 1 unclear

As the focus of this study was on the experiences of the users—older migrant patients and health professionals—of digital translation tools in transcultural care interactions, the presentation of the results does not evaluate each translation tool specifically. Instead, the tools are mentioned in general terms based on the experiences using them. Three main themes and eight subthemes emerged from the analysis (Table 2): *theme one* describes experiences where the users felt that the digital translation tool supported communication during interactions between patients and health professionals; *theme two* describes experiences of the limitations encountered when using the digital translation tool; and *theme three* describes suggestions for improvements to the digital translation tools.

**Table 2.** Description of Themes and Subthemes.

Themes	Subthemes
Advantages of using translation tools	Participating in one’s own care
	An aid to overcoming language barriers in daily interactions
Limitations and challenges	User challenges
	Limitations in translation tool functionality
	Limitations of the work environment
Improvement suggestions for the functions of the translation app	Adaptations to older users
	Improvement suggestions from health professionals’ perspectives
	Accessibility and patient safety

## Advantages of Using Translation Tools

The older patients described their experiences using translation tools from both social and emotional perspectives, how they felt using the tool in their interactions with health professionals, and the advantages of the translation tools’ various functions.

### Participating in One’s Own Care

Older migrant patients described how the digital translation app helped them focus on the conversations with health professionals, enabling active participation in their own medical treatment and care (Freyne et al., 2018). Everyday requests were seen as important, such as asking for help going to the toilet, taking medication, getting a glass of water, or communicating about pain and other symptoms related to their health condition (Hwang et al., 2022; Silvera-Tawil et al., 2021; Yost et al., 2009). Health professionals, on the other hand, described it as satisfying to be able to provide appropriate and patient-safe care when they did not have to guess patients’ care needs (Freyne et al., 2018; Silvera-Tawil et al., 2021).

### An Aid to Overcoming Language Barriers in Daily Care Interactions

The translation tool was seen as helpful in overcoming language barriers in simple conversations in care interactions. The health professionals used it to identify pain; to assist in showering/dressing, eating and drinking, and medical administration; to give safety messages; for positioning and orientation; for making observations and facilitating instructions, demonstrations, and explanations; for explaining medication use, obtaining consents; skin checks; and procedures such as catheter, canula, or intubation insertion or removal (Freyne et al., 2018; Hwang et al., 2022; Panayiotou et al., 2020; Silvera-Tawil et al., 2018, 2021; Tatemoto et al., 2021; Yilmaz et al., 2020). Older migrant patients believed it was valuable to be able to communicate directly with the health professionals without having a family member present (Panayiotou et al., 2020). During the COVID-19 pandemic, communication between patients and health professionals had to be conducted digitally. One older patient had to navigate a rehabilitation program by using a translation tool, and understood the instructions and was able to successfully participate (Tatemoto et al., 2021). Generally, the digital translation tools were described as “easy to use” and the questions and phrase libraries were perceived as “interesting” (Hwang et al., 2022; Panayiotou et al., 2020). They were even considered educational and a source of information for health-related issues (Yost et al., 2009). Health professionals described the digital translation tool as time-saving, because they could communicate directly with patients without having to wait for a human interpreter (Panayiotou et al., 2020).

The translation app was also considered useful in areas other than care interactions, such as in administrative tasks like health care documentation, hospital admission, and discharge (Hwang et al., 2022; Silvera-Tawil et al., 2018). Nurses described the functionality of the digital translation apps as particularly advantageous when translation was both written and oral and when the text was clarified with images and illustrations (Freyne et al., 2018; Hwang et al., 2022; Panayiotou et al., 2020; Silvera-Tawil et al., 2018, 2021; Yost et al., 2009). Health professionals considered it advantageous to have phrase libraries as a base for the conversations during needs assessments or standardized pain assessments, which were considered easier to document (Hwang et al., 2022; Silvera-Tawil et al., 2018, 2021).

## Limitations and Challenges

Despite older migrant patients and health professionals considering the digital translation app to be helpful in simple everyday conversations, there were limitations among users as well as limitations in the functionality of the translation tools and the work environment.

### User Challenges

Some older migrant patients found the translation tool difficult to use, describing their unfamiliarity with technology resulting in a longer learning time compared to those familiar with technology (Panayiotou et al., 2020; Yilmaz et al., 2020; Yost et al., 2009). Some health professionals also expressed discomfort using technology and were embarrassed about not knowing how to use the digital translation app in front of patients (Silvera-Tawil et al., 2021). Nurses also perceived that the translation app was not suitable for patients with cognitive impairments, confusion, or dementia (Hwang et al., 2022; Yilmaz et al., 2020).

### Limitations in Translation Tool Functionality

Patients and health professionals found it to be frustrating when the translation was inaccurate and when language dialects were not translated correctly. When there were missing phrases for what patients wanted to express or when patients could only provide structured responses—that is, select limited response options to structured questions—health care conversations were perceived as limited (Hwang et al., 2022; Panayiotou et al., 2020). A serious incident was when one translation tool was used to diagnose an older patient’s symptoms who was then sent back home. When being re-admitted to the hospital, an authorized translator was used, and the older migrant patient was able to describe the symptoms of his condition in more detail. The further investigation, revealed he had cancer (Leyden & Melby, 2021). Some patients also described difficulty understanding the medical vocabulary (Panayiotou et al., 2020; Yost et al., 2009). Health professionals perceived that health care conversations were limited and could only be held at a basic level. In more advanced conversations, such as a doctor explaining a diagnosis or in situations involving terminal illnesses, authorized translators should be used (Freyne et al., 2018; Panayiotou et al., 2020; Silvera-Tawil et al., 2018, 2021).

### Limitations of the Work Environment

Other limitations of translation tools were described as being related to the actual work environment and organization, such as a limited or unstable internet connection (Hwang et al., 2022; Panayiotou et al., 2020); the availability of digital tools being too few in number or located too far away from the patient (Freyne et al., 2018; Silvera-Tawil et al., 2021); or that digital tools used by all health professionals and patients could be a risk factor for infection transmission (Silvera-Tawil et al., 2018).

## Improvement Suggestions for the Functions of the Translation App

Based on the limitations described by the participants in the studies, migrant patients and health professionals provided suggestions for improvements and adaptations to the digital translation apps to make them more user-friendly and easier to use for older adults and technology-savvy individuals in general.

### Adaptations to Older Users

For the older users, the functions and usability were important. In cases of hearing impairment, older migrant patients suggested that the audio function should be adjustable, allowing individuals with hearing impairments to adjust the volume or use headphones or an amplifier to increase the sound volume (Panayiotou et al., 2020; Silvera-Tawil et al., 2021). For those with vision impairments, it was important to be able to enlarge the letters of the texts, so they could see the text message (Silvera-Tawil et al., 2018). Another improvement suggestion was an adjustable speech rhythm, as older migrant patients described that the speech was sometimes too fast and they needed to hear the text multiple times (Silvera-Tawil et al., 2021).

Further improvement suggestions included availability in multiple languages and the inclusion of more phrases for nuanced conversations, such as describing or explaining different conditions or kinds of pain (Freyne et al., 2018; Panayiotou et al., 2020; Silvera-Tawil et al., 2021). There was also a desire among older migrant patients for less medical language in the tools, as they did not always understand the meaning of the medical words. A calendar function to remind patients of upcoming appointments and procedures was another suggestion (Silvera-Tawil et al., 2018, 2021; Yilmaz et al., 2020).

### Improvement Suggestions From Health Professionals’ Perspectives

Health professionals believed that pictures and illustrations were good for clarifying the meaning of questions and text messages (Silvera-Tawil et al., 2018), but also suggested including video materials to demonstrate procedures and instructions in the translation app (Freyne et al., 2018; Yilmaz et al., 2020). It was also suggested that phrase libraries should be adjustable according to health disciplines, as a way to ask questions or speak with the patients which could involve specific discipline-related words and structure versus open questions. A dietician may, for example, want open responses, while a physiotherapist may want to use more structured questions (Freyne et al., 2018). Health professionals also suggested that the translation apps should be more culture- and gender specific, such as including both male and female voices (Hwang et al., 2022; Silvera-Tawil et al., 2018; Yilmaz et al., 2020).

Health professionals proposed that education on how to use the translation tools would be beneficial and should be provided by the employer (Panayiotou et al., 2020).

### Accessibility and Patient Safety

Other improvement suggestions concerned the accessibility, placement, hygiene, and patient safety of digital tools. The health professionals suggested that the translation tools should be placed close enough to the patient and that they should be easily accessed in different care situations; for example, by the patient’s bed (Freyne et al., 2018; Silvera-Tawil et al., 2021). The translation tool should also be easy to keep clean to avoid becoming a “vector of infection,” as it is used by several patients and health professionals (Silvera-Tawil et al., 2018). Furthermore, as the conversations between patients and health professionals must be confidential, the apps used must be designed to guarantee patient safety (Yilmaz et al., 2020).

## Discussion

The results showed that both older migrant patients and health professionals experienced several advantages in using translation tools in health care interactions. Patient experiences revolved around how they perceived the translation tool’s impact on their relationship with health professionals, which was mostly positive. Even though most of the included studies agreed that the use of a digital translation tool was helpful in basic care situations, they should not be used as a substitute for authorized human interpreters, especially when it comes to longer and more complex conversations. However, the seemingly simple conversations through which patients and health professionals could understand each other through the help of the translation tool could contribute to building trusting relationships, which in turn affect treatment compliance and patients’ experience of care (Mangrio & Sjögren Forss, 2017). Being able to speak for oneself in various health care situations could contribute to a sense of participation, which is in line with earlier research that supports patients’ involvement in their own decision-making regarding treatment and care (Radl-Karimi et al., 2020).

Health professionals who are able to provide care based on mutual understanding of patients’ needs minimizes the risk of misunderstandings and can prevent care injuries (Chang et al., 2021; Flores et al., 2012). The time-saving aspect was appreciated by health professionals, who, in having direct access to a digital translation tool, did not need to wait for a human interpreter. This is important in emergency situations which require immediate intervention.

However, there were limitations to the use of the translation apps. One was the older users’ lack of familiarity with technology; older patients and health professionals of various ages experienced this to be a barrier. In the use of technical and digital tools, the perspectives of older users are important, as aging itself brings about both physical and cognitive changes (Wandke et al., 2012). The concerns of the older migrant patients included the functions of the digital translation apps, as the functions of the apps were not necessarily adapted for older users; particularly not those who had various cognitive conditions. Digital and technical tools instead may increase confusion and create anxiety. An interesting contrast is that many patients admitted to hospitals today are older, but the digital devices used are not necessarily tailored for these age groups. The design of technical devices should be based on a larger variation of the users.

Practical aspects, like the accessibility of the digital translation tools, hygiene, and patient safety should be considered when implementing such devices in hospital wards. Patient data must be kept confidential, and the tools used should guarantee confidentiality. Even though the themes “improvements,” like visual or hearing aids for older patients, or availability of the correct concepts for professionals, and “limitations” such as functionality or environment, describe aspects of care, digital tools may enhance or present an obstacle to care communication, which may be explained by considering health literacy. Being able to use digital technology, whether a patient or a health professional, is part of digital health literacy in the care environment. When the ability to use digital tools as means to communicate and present the ability for professionals to offer high-quality care, it can decrease risks if adapted to different professions and the specific concepts used by that profession. To the older patient, health literacy—may offer empowerment and autonomy in aging, especially in vulnerable situations of health problems or diseases. Relevant areas for implications can for example; enhance communication and equity in digital health information, training of clinicians on communication, and awareness of migrants with limited access and use of information, and development of strategies that consider migrants’ needs (WHO, 2013).

With proper language and communication skills, the patient and the health care professionals can understand each other and the actions taken can be mutually agreed upon.

In contrast to popular opinions of older adults’ unwillingness of using technology, earlier studies have shown (Kim et al., 2022; Offerman et al., 2024), as well as in this study that older adults are willing to use digital tools but that physical challenges related to aging may be a barrier. Constructors of translation tools need to bear in mind that digital translation tools should be adjusted to the needs and abilities of older ages. The results also indicated that the cultural contexts in which digital translation tools are used should be considered. Although language is only part of a culture, it is a mediator of the traditions, values, and views which defines a cultural context and influences the individuals of a culture

Further studies on the perspectives of older users are needed, as well as studies following up the use of the translation tools in care interactions over time.

## Limitations of the Study

A limitation in this literature review is that it is based on only a few articles, which may be due to narrow inclusion/exclusion criteria. If the criteria had been broader, perhaps other types of digital communication tools, more articles may have been found, but since the aim of this study was specifically to explore experiences of using translation tools in transcultural care, the number of articles was assessed as acceptable. The inclusion of case studies here can be questioned, but since they contain rich descriptions of care interactions, it was assessed that they contributed to understanding of the phenomena. In terms of scientific quality assessment most of the included articles were unclear about the researcher’s role, as this can be an aspect of trustworthiness. The method descriptions in the included articles, except one case-study, were well described, which enhances trustworthiness of the results.

## Conclusions

The experiences of the users of these translation tools varied from perceiving the tools as a complement in care interactions. For the older migrant patients to build trusting care relationships, whereas health professionals’ confidence increased in knowing that care was based on mutual understanding and including rather than excluding the migrant patients from care decisions. Translation tools may be used in basic care situations, but should not be a replacement for human interpreters. The need to understand individuals with other ethnic backgrounds and languages still seems to require professional human interpreters.
